# Case-control study: Determination of potential risk factors for the colonization of healthy volunteers with *Streptococcus gallolyticus* subsp. *gallolyticus*

**DOI:** 10.1371/journal.pone.0176515

**Published:** 2017-05-01

**Authors:** Jessika Dumke, Tanja Vollmer, Oke Akkermann, Cornelius Knabbe, Jens Dreier

**Affiliations:** 1 Institut für Laboratoriums- und Transfusionsmedizin, Herz- und Diabeteszentrum Nordrhein-Westfalen, Universitätsklinik der Ruhr-Universität Bochum, Bad Oeynhausen, Germany; 2 Johannes Wesling Klinikum Minden, Minden, Germany; Universidade de Lisboa Faculdade de Medicina, PORTUGAL

## Abstract

*Streptococcus gallolyticus* subsp. *gallolyticus* was identified in humans and animals as commensal of the gut and can act as a causative agent of endocarditis and septicemia. A case-control study was performed to identify yet unknown risk factors for the transmission of this facultative pathogen. The prevalence in the gut of 99 healthy volunteers was determined using real-time polymerase chain reaction resulting in 62.5% *S*. *gallolyticus* subsp. *gallolyticus* positive excrements. Subsequent cultivation offered three isolates and epidemiological analysis based on MLST revealed sequence type (ST) 3 and ST 7, previously detected from bovine and endocarditis patients. These results support the hypotheses of the zoonotic potential of this bacterium. Participant questionnaires were evaluated concerning personal characteristics, nutritional habits and animal contact. Specifically, closer contact between participants and animals influenced the colonization of the human gut significantly and was further affected if volunteers used excrement for the fertilization of plants.

## Introduction

*Streptococcus gallolyticus* subsp. *gallolyticus*, formally known as *Streptococcus bovis* biotype I, belongs to the Lancefield group D Streptococci [1], is a normal inhabitant of the animal and human gastrointestinal tract, and appears in 2.5 to 15% of healthy humans [2]. On the contrary, its frequency in the digestive tract of animals and the absolute frequencies in various species are not well described. To date, there is only one study, which estimated the percentage of *S*. *gallolyticus* subsp. *gallolyticus* in feces of turkeys. The detection rate in fecal samples of turkeys is 91% [3]. It was also identified in pigeon, bovine and chicken as commensal bacterium [4–6]. However, *S*. *gallolyticus* subsp. *gallolyticus* can also act as a facultative pathogen, causing sepsis, meningitis and infective endocarditis (IE) in humans and animals [6–8]. Human IE is especially associated with colorectal cancer [9–11]. The incidence of group D *Streptococcus*-associated diseases is increasing in the south of Europe [12]. The detection in humans and animals as a causative agent producing the same clinical symptoms leads to the assumption that *S*. *gallolyticus* subsp. *gallolyticus* may be a zoonotic pathogen [13]. Investigations in France and Spain suggest a correlation between a rural residency and the presence of the facultative pathogen [14,15]. The transmission of the potential zoonotic pathogen may be directly by smear or droplet infections or indirectly from surfaces contaminated with *S*. *gallolyticus* subsp. *gallolyticus* [16]. The transfer of the bacterium through a closer contact with colonized or infected animals is also discussed as a possible mechanism [13,14]. It was described as an important risk factor for the transmission of *Streptococcus suis* between infected animals and humans [17,18]. Epidemiologic analyses in a laying hen flock in North Rhine Westphalia also contribute to the assumption that a closer occupational contact with colonized laying hens may be a potential risk factor for the colonization of the gastrointestinal tract with *S*. *gallolyticus* subsp. *gallolyticus*, since the bacterium was identified as the causative agent of IE of the farm owner [13]. In addition to the detection of *S*. *gallolyticus* subsp. *gallolyticus* in eukaryotic organisms, it was also identified in milk and raw milk products (especially in dairy cows with mastitis) and red meat [14,19–21]. The detection in food leads to the assumption that the transmission of *S*. *gallolyticus* subsp. *gallolyticus* between animals and humans can be connected to dietary habits. Exemplarily, *Streptococcus equi* subsp. *zooepidemicus* was transmitted through the consumption of unpasteurized raw milk in an outbreak setting [22]. There have been no investigations to date which systematically analyze the correlation between dietary habits or the contact with animals and the detection of *S*. *gallolyticus* subsp. *gallolyticus* in the human gut. Therefore, we conducted an epidemiological study which is comprised of two parts: Firstly, the case-control study to determine the prevalence of *S*. *gallolyticus* subsp. *gallolyticus* and the associated risk factors for the colonization of the human gut, and secondly, multilocus sequence typing (MLST) to characterize the *S*. *gallolyticus* subsp. *gallolyticus* population structure. This analysis identified a correlation between lifestyle habits and the human gastrointestinal colonization with *S*. *gallolyticus* subsp. *gallolyticus*.

## Material and methods

### Sample and data acquisition

A retrospective case-control study was conducted at the Herz- und Diabeteszentrum Nordrhein-Westfalen (Bad Oeynhausen, Germany) from December 2012 to July 2015. The case-control study used word of mouth to recruit people. All data are collected in pseudonymous form. A total of 135 volunteers from the north and west of Germany participated in this study. Written consent was required for the case-control study. Fecal samples were tested and a questionnaire was completed by each volunteer to analyze the correlation between the fecal presences of *S*. *gallolyticus* subsp. *gallolyticus* and potential risk factors. Furthermore, seven SGG-culture-positive tested healthy volunteers were selected and were analyzed two to three times to estimate the gastrointestinal presence of *S*. *gallolyticus* subsp. *gallolyticus* in a follow-up period (follow-up study). Participants were excluded from the study if there was no fecal sample, no completed questionnaire or no written consent. In addition, only healthy volunteers (without gastrointestinal diseases or IE) over 18 years were included for the identification of risk factors. These data were received from the questionnaires. People were also excluded if an antibiotic therapy was indicated six months prior to participation.

The study was approved by the ethics commission of the Ruhr University Bochum Faculty of Medicine.

### Stool investigations

#### DNA extraction

DNA extraction of homogenized fecal samples was performed by using NucliSENS easyMAG (Biomerieux, Nürtingen, Germany). DNA extraction was generally performed according to the manufacturer’s instruction. The fecal samples were pretreated by inoculating material (about 0.1 g) in 1 ml PBS in a tube with Zirconia beads, mixed for 5 min, incubated for 10 min at room temperature and then centrifuged at 12000 × *g* for 2 min. A quantity of 200 μl of the supernatant was used for the extraction of the whole DNA. After prelysis within the NucliSENS easyMAG, 100 μl magnetic silica particles were added and extraction was performed as described by the manufacturer. DNA was eluted in 55 μl elution buffer.

#### Real-Time PCR

The detection of an internal fragment of the *recN* gene was used to screen fecal specimens for the detection of *S*. *gallolyticus* subsp. *gallolyticus* [23]. The PCR amplification for the presence or absence of this gene was carried out within a 50 μl reaction volume containing 5 μl template DNA, 5 μl Platinum-*Taq*-buffer (ThermoFisherScientific, Darmstadt, Germany), 200 nM of each primer (F-recN SGG/P: 5’-GATTTTCAAGTCCAATTCACCAAAG-3’, R-recN SGG/P: 5’-GGTTYGTTGAAATGTAAAATTCAACAG-3’; LifeTechnologies, Darmstadt, Germany), 100 nm of the Pf-recN/SGG-probe (5’-FAM-TTCAATCGTGATGGCAA-MGB-3’; LifeTechnologies, Darmstadt), 240 μM dNTPs (Fermentas, Leon-Rot, Germany) and 0.25 μl Platinum-*Taq*-polymerase (ThermoFisherScientific, Darmstadt). The detection of *S*. *gallolyticus* subsp. *pasteurianus* was performed using the Pv-recN/SGP-probe (5’-VIC-TCAACCGTGATGGAAA-MGB-3’) and the same primers denoted above [23]. The internal control used in the reaction mix was CMV-DNA (CMV-TM2-F: TTYTTAGCACGGGCCTTAGC, CMV-TM2-R: AAGGAGCTGCATGATGTGASC; CMV-TM2-S: CY5-TGCAGTGCACCCCCCAACTTGTT-BHQ2; [24]). Diluted DNA extracted from a bacterial overnight culture of *S*. *gallolyticus* subsp. *gallolyticus* (ATCC BAA-2069) or *S*. *gallolyticus* subsp. *pasteurianus* (DSM 15351) was used as positive control and water as negative control to verify the specificity of the PCR reaction. A two-step PCR on the Rotor Gene Q platform (Qiagen, Hilden, Germany) was performed. Amplification of PCR products was carried out as follows: initial denaturation at 95°C (5 min) followed by 50 cycles, and a denaturation 95°C (15 s), annealing and elongation step at 60°C (60 s).

#### Selective cultivation

Real-time PCR *S*. *gallolyticus* subsp. *gallolyticus* positive-tested fecal samples were further selectively cultivated on modified trypton soya agar (TSA) (0.5% tannic acid pure (AppliChem GmbH, Darmstadt, Germany, 0.25 g/l sodium acetate, Merck, Darmstadt, Germany), as described previously [3]. Briefly, the homogenized fecal sample was streaked out onto selective medium before weighing and suspending in PBS buffer. Then, 1 g of homogenized fecal sample was suspended in 1 ml PBS medium, mixed and streaked out with PBS (duplicate) and 100 μl was plated as triplicate onto sodium acetate tannic acid TSA. It was then incubated at 37°C and 5% CO_2_ for 48 h [3]. In parallel, an overnight grown culture of *S*. *gallolyticus* subsp. *gallolyticus* was plated onto modified TSA. Single putative *S*. *gallolyticus* subsp. *gallolyticus* colonies were selected and analyzed regarding species and subspecies level by matrix-assisted laser desorption ionization—time of flight mass spectrometry (MALDI-TOF-MS) and *sodA* sequencing [25].

### Multilocus sequence typing

Multilocus sequence typing was performed, as described preciously [16]. In brief, the total DNA of *S*. *gallolyticus* subsp. *gallolyticus* isolates was isolated by using a QIAamp Blood Mini Kit (Qiagen, Hilden, Germany) and 5 μl was used for each fragment amplification [16]. Partial sequences of the housekeeping genes *aroE*, *glgB*, *nifS*, *p20*, *tkt*, *trpD* and *uvrA* were amplified, sequenced and analyzed [16]. All detailed protocols can also be found on www.pubmlst.org [16]. The determination of sequence types (STs) was undertaken using the pubMLST database and Bionumerics Software 6.6 (Applied Maths, Sint-Martens-Latem, Belgium) [16,26]. For the characterization of the strains a minimum spanning tree was generated and eBURST version 3 (based upon related sequence types; www.mlst.net) was used to calculate clonal complexes [16,27].

### Questionnaire

The questionnaire included 25 questions and sought data on the following aspects: personal characteristics (age, gender, gastrointestinal diseases, residence [urban, rural, landscape—near the forest/farm] and antibiosis), contact with animals (living or working on a farm, private or occupational contact) and dietary habits (consumption and handling of minced meat, raw milk or raw milk products). The exposure factors as well as the absolute frequencies can be found in S1 Table.

### Statistical analysis

The statistical analysis software SPSS version 21 was used. Binary logistic regression was utilized to establish a model to determine the simultaneous influence of potential risk factors. Six independent variables (age, gender, consumption of raw animal products, close animal contact, usage of animal waste) were tested within the multiple logistic regression model to verify adjusted odds ratios (ORs). Statistical tests were considered to be significant if the p-value was less than 0.05. A confidence interval of 95% was used for both calculations. Forest plots were generated using Microsoft Excel. The age was listed as mean plus/minus standard deviation.

## Results

A total of 134 participants (65 men and 69 women with a mean age of 48.4 ± 14.9 years) were included in the case-control study. After the application of exclusion criteria, 99 healthy volunteers were included to identify potential risk factors for the colonization of the human gut with *S*. *gallolyticus* subsp. *gallolyticus*. The fecal samples of healthy volunteers were screened by PCR for the presence of the facultative pathogen. *S*. *gallolyticus* subsp. *gallolyticus* was detected in 62.5% (n = 59) of the 99 fecal specimens of healthy volunteers. The presence of *S*. *gallolyticus* subsp. *pasteurianus* was also estimated using the VIC labeled probe in the PCR reaction mix. This bacterium was detected seven times out of 99 volunteers. Real-time PCR testing also identified three subjects who were colonized with *S*. *gallolyticus* subsp. *gallolyticus* as well as *S*. *gallolyticus* subsp. *pasteurianus* simultaneously. These volunteers are recognized in the *S*. *gallolyticus* subsp. *gallolyticus*-positive group.

*S*. *gallolyticus* subsp. *gallolyticus* PCR-positive specimens were cultured on selective medium to isolate this bacterium for epidemiologic characterization by MLST. Three isolates, namely HDZ 1323, HDZ 1330 and HDZ 1332, were detected by culture and mass spectrometric analyses, and *sodA* sequencing confirmed *S*. *gallolyticus* subsp. *gallolyticus*. In addition to *S*. *gallolyticus* subsp. *gallolyticus*, three out of seven *S*. *gallolyticus* subsp. *pasteurianus* isolates were identified by MALDI-TOF MS and sequencing of the partial fragment of the *sodA* gene. In this regard, the real-time PCR demonstrated one inconsistent result: *S*. *gallolyticus* subsp. *gallolyticus* was detected instead of *S*. *gallolyticus* subsp. *pasteurianus*.

The *S*. *gallolyticus* subsp. *gallolyticus* isolates selected were further typed using MLST. It revealed the sequence types ST 3 (HDZ1330), ST 7 (HDZ1323) and the newly defined ST 105 (HDZ1332). Bionumerics Software 6.6 was used to utilize for the construction of a minimum spanning tree (S1 Fig). The minimum spanning tree of the strains revealed no phylogenetic relationship of these detected STs (S1 Fig). The allelic profiles (STs) show no identical allelic numbers within the identified sequence types of the case control study (one exception: the number of the *nifS* allele from ST 3 and 7). The ST 3 was already identified in human heart valve cultures and from the intestine of a bovine, which was also detected for ST 7 isolates ([16], www.pubmlst.org).

In order to identify the *S*. *gallolyticus* subsp. *gallolyticus* status in the gastrointestinal tract over time, a follow-up investigation of seven culture positive tested healthy volunteers was performed until the end of the study (a total of 2 to 3 samples per person) (Table 1). Initially, six fecal specimens were screened as real-time PCR positive for *S*. *gallolyticus* subsp. *gallolyticus* and one sample was tested as positive for *S*. *gallolyticus* subsp. *pasteurianus*. Selective cultivation offered three *S*. *gallolyticus* subsp. *gallolyticus* and one *S*. *gallolyticus* subsp. *pasteurianus* isolate. Further analyses of specimens revealed *S*. *gallolyticus* subsp. *gallolyticus* in four cases at any tested time point by using real-time PCR (volunteer 4 to 7). As an example, the first (January 2015) and second sample (March 2015) were tested as positive and the third specimen in July 2015 was tested as negative (volunteer 3). The fecal sample of the 7th volunteer was initially tested as positive (April 2013) and the second sample 26 months later was detected as positive for the presence of *S*. *gallolyticus* subsp. *gallolyticus* (Table 1). At least one exception was identified. The PCR results of the second sample from volunteer 1 showed the presence of both subspecies, and *S*. *gallolyticus* subsp. *pasteurianus* was isolated using modified trypton soya agar (Table 1).

**Table 1:** Follow-up study of healthy volunteers.

	Fecal sample 1		Fecal sample 2		Fecal sample 3	
	Real-time PCR	Selective cultivation	Real-time PCR	Selective cultivation	Real-time PCR	Selective cultivation
	Sample date		Sample date		Sample date	
Volunteer 1	SGP	SGP	SGP, SGG	SGP	-	
	December 2014		March 2015			
Volunteer 2	SGG	SGG	negative	negative	negative	negative
	November 2014		March 2015		July 2015	
Volunteer 3	SGG	SGG	SGG	negative	negative	negative
	January 2015		March 2015		July 2015	
Volunteer 4	SGG	SGG	SGG	negative	SGG	negative
	December 2014		March 2015		July 2015	
Volunteer 5	SGG	Negative	SGG	Negative	-	
	November 2014		March 2015			
Volunteer 6	SGG	negative	SGG	negative	SGG	negative
	December 2014		March 2015		-	
Volunteer 7	SGG	negative	SGG	negative	-	
	April 2013		Juni 2015			

SGG: *S*. *gallolyticus* subsp. *gallolyticus*; SGP: *S*. *gallolyticus* subsp. *pasteurianus*

**Fig 1:**
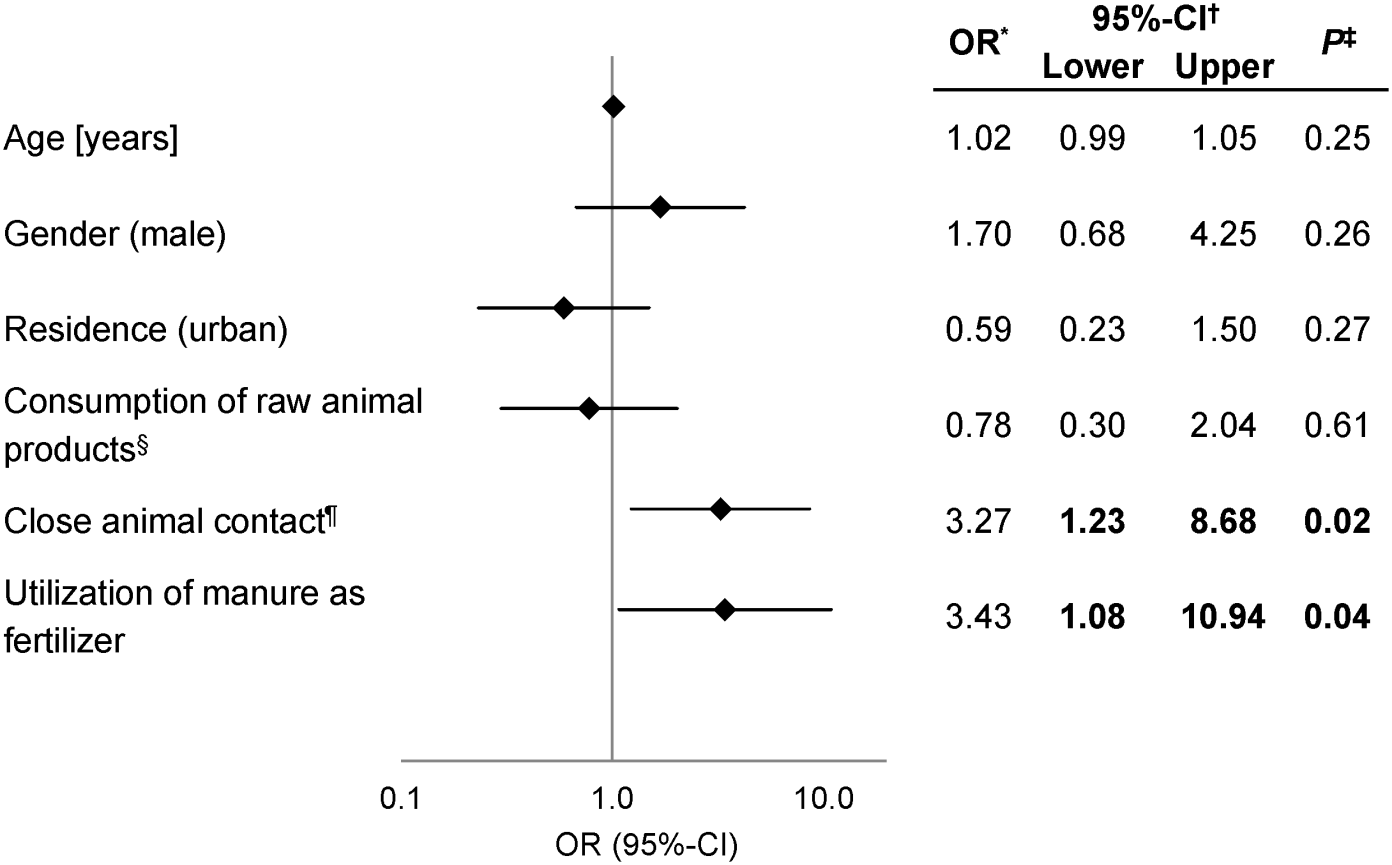
Binary logistic regression model of exposure factors for the colonization of the human gastrointestinal tract. Potential risk factors discussed in the literature were included in the logistic regression model and adjusted ORs* were calculated with a confidence interval (CI^**†**^) of 95%. The x-axis is displayed logarithmically. *P*-values were calculated using the chi-square test (^**‡**^). Results were significant if a *p*-values less than 0.05 was detected or the 95%-CI does not include 1 and were indicated in **bold**. ^**§**^ raw minced meat, raw milk and raw milk products; ^**¶**^ Contact with excrement or saliva of animals, striking of animals.

Based on the real-time PCR detection of the bacterium in fecal samples, cases and controls were defined: 59 cases (male/female [m/f] ratio: 25/34) and 40 controls (m/f ratio: 21/19). The cases included an age distribution from 20 to 70 years with a mean value of 44.2 ± 14.6 years and controls from 22 to 82 years with an average of 49.2 ± 15.0 years. The cases and controls were analyzed in terms of their nutrition habits, contact with animals and residence (rural, urban; forest or farm next to their residence). The frequencies of potential risk factors observed in cases and controls are listed in S1 Table. Logistic regression was performed to analyze the simultaneous effect of risk factors and adjusted ORs were calculated and presented as a forest plot (Fig 1).

Multiple expositions often characterize the outcome of a disease or different event such as the *S*. *gallolyticus* subsp. *gallolyticus* colonization of the human gastrointestinal tract. Adjusted ORs were calculated to assess the simultaneous effect of the variables. A closer animal contact between volunteers and animals (OR: 3.27, CI: 1.23–8.68; *p* = 0.02) and the usage of animal waste to fertilize plants (OR: 3.43, CI: 1.08–10.94; *p* = 0.04) demonstrate significant risk factors for the transmission between animals and humans and to colonize the gastrointestinal tract of healthy people (Fig 1).

In conclusion, simultaneous testing of exposure factors indicate a higher risk of being colonized with *S*. *gallolyticus* subsp. *gallolyticus* of participants who have a direct contact with animals and utilization of manure. Furthermore, two out of three *S*. *gallolyticus* subsp *gallolyticus* isolates reveal the STs 3 and 7. These STs were previously isolated from human blood cultures and bovine ([16], www.pubmlst.org).

## Discussion

The knowledge of transmission pathways and the zoonotic potential of the facultative pathogen *S*. *gallolyticus* subsp. *gallolyticus* are quite limited. Thus, a systematic approach was conducted for the first time to determine the latter’s occurrence in the gut of healthy people and to describe the risk factors for the transmission of the bacterium and its colonization of the human gastrointestinal tract. *S*. *gallolyticus* subsp. *gallolyticus* is an opportunist of the gastrointestinal tract in humans and animals with varying prevalence in the healthy human population of 2.5 up to 15% [2], but the *S*. *bovis* fecal carriage increased three to five times in patients with colorectal cancer and inflammatory bowel disease [8,10,28,29]. In comparison to the previous studies real-time PCR screenings of fecal samples offer a much higher prevalence in healthy volunteers of 62.5%. Spanish real-time PCR investigations of patients who underwent colonoscopy revealed 11.1% *S*. *gallolyticus* subsp. *gallolyticus*-positive and 13% *S*. *gallolyticus* subsp. *pasteurianus*-positive rectal swabs. It indicates a similar portion of both subspecies in the gastrointestinal tract [23], which cannot be confirmed by real-time PCR screenings of feces from participants of the case-control study. Similar results were detected for the presence of *S*. *gallolyticus* subsp. *pasteurianus*. The detection of both subspecies using real-time PCR may indicate a co-occurrence in the digestive tract, which was also suggested by Lopes *et al*. [23].

The divergences observed between previous studies may be a resumé of the sample sets analyzed (colonoscopy [30], feces [28], rectal swabs [23]) or the kind of screening methods (cultivation [30,31], molecular techniques [23,32]) to identify or isolate *S*. *gallolyticus* subsp. *gallolyticus*. A higher sensitivity of the molecular screening method was demonstrated by positive real-time PCR results in comparison to selective cultivation and was also confirmed in the follow-up study. The complexity and characteristics of the sample type and difficulties in homogenization as well as the gut microbiota may influence the PCR results (e. g. inhibitors) and the selective cultivation [33]. It explains not only false negative real-time PCR results, but also discrepancies between *S*. *gallolyticus* subsp. *gallolyticus*-positive culture and the *S*. *gallolyticus* subsp. *pasteurianus*-positive real-time PCR screening results. As suggested in a 17-year follow-up study [30], the prospective investigations of seven participants achieved shifts in the composition of the gut microbiome, which was e. g. demonstrated for the participant three. Nutrition and medicines shape the gut microflora (e. g. antibiotics), whereas antibiotics change the gut composition up to one year [34–36]. This may also be transferred to our follow-up study. Although the participants with an antibiotic therapy were excluded it is not known if the composition of the gut microbiome affects the presence of *S*. *gallolyticus* subsp. *gallolyticus* in the gut. However, the sample age may be of more importance than the sample characteristics, microbiota or processing. The fecal samples were sent to the laboratory by mail. Consequently, samples could be in transit for three days before processing in the laboratory. Although a survival of *S*. *gallolyticus* subsp. *gallolyticus* was demonstrated *in vitro* in *S*. *gallolyticus* subsp. *gallolyticus*-negative tested human stool specimens for 14 d at RT (20°C) (real-time PCR and cultivation; data not shown) it cannot be ruled out, that the growth of other gastrointestinal bacteria may inhibit growth of *S*. *gallolyticus* subsp. *gallolyticus* or a less concentration of *S*. *gallolyticus* subsp. *gallolyticus* in the feces may effect a false negative cultivation. It is assumed that the presence of the same diseases in animals and humans may be a hint that *S*. *gallolyticus* subsp. *gallolyticus* is a zoonotic pathogen [16]. This was supported by MLST, which typed a blood culture isolate of an animal farmer and excrements of the chicken of his laying hen farm with the same ST [13]. Interestingly, the human fecal isolates which were identified in this case-control study were differentiated into the STs 3 and 7 and the new identified ST 105. ST3 and 7 were associated with human blood culture isolates and were also identified in cattle (unknown infective status) [16]. Thus, these results support the potential transmission of *S*. *gallolyticus* subsp. *gallolyticus* between animals and humans and highlight the zoonotic potential of the facultative pathogen. As described previously, it might be the case, as there seems to be some STs (as is the case of ST 7 or ST 3) that seem more associated with humans or animals whereas other isolates are more predominant in animals and humans (e. g. isolates of the clonal complex 45 and 6) [16]. A prevalence of 62.5% in human feces described in this study and a high prevalence in organic turkey flocks give rise to the question if *S*. *gallolyticus* subsp. *gallolyticus* belongs to the common gut microbiome of animals and humans [3]. However, for this conclusion further systematic analyses have to be performed.

Among other diseases, *S*. *gallolyticus* subsp. *gallolyticus* causes IE and is associated with colorectal diseases in humans [10,11,37–40]. Both diseases are generally more often observed in male patients over 50 years old [41,42] and various studies demonstrated the same positive association between the isolation of *S*. *gallolyticus* subsp. *gallolyticus* in men and the elderly population and IE [15,43–47], which was not identified in this study (Fig 1). To identify a relationship between the one-year increasing age and the detection of the bacterium in the digestive tract more people have to be tested.

The facultative pathogen is the most frequently detected agent in cases of infective endocarditis in rural regions (especially in the cattle and milk production area) in the south of Europe [12,14,15], which cannot be observed in this case control study (Fig 1) and should be figured out in further investigations. Joined together with living in the countryside, a close contact with animals was supposed to be a transmission source of the bacterium [8,14], it was demonstrated for the first time that a closer contact with animals is a significant exposure factor to be colonized with *S*. *gallolyticus* subsp. *gallolyticus*. It can be assigned as risk factors for the transmission of *S*. *gallolyticus* subsp. *gallolyticus* between animals and humans and its establishment in the human gut. Another interesting fact is the kind of animal species which come into contact with people. Although dogs and horses are described in the literature as a source of isolation and are common pets [1,5], it is not known whether these animals belonging to the volunteers are colonized with the bacterium. In this context, it is remarkable that fertilization of plants with the excrement of animals increases the risk of carrying *S*. *gallolyticus* subsp. *gallolyticus* significantly. Consequently, it is imaginable that *S*. *gallolyticus* subsp. *gallolyticus* may be transferred directly from animals to humans by smear infections and colonizes the gastrointestinal gut and, thus, can be accounted as a significant risk factor. However, the prevalence of *S*. *gallolyticus* subsp. *gallolyticus* in animals is still unknown [3]. The bacterium has often been identified in, for example, pigeons, chicken and turkeys [3–6,13,48,49]. Therefore, future studies should also include investigations of animal excrement in addition to human specimens to verify the prevalence in animals, too, and to estimate the real risk to human health.

Derived from this hypothesis, not only the animal contact, but also the consumption of or the contact with contaminated food, such as red meat or milk products, may promote the colonization of the human gut and are propagated as exposure factors for the transmission of the bacterium from animals to humans [15]. In total, statistical analyses demonstrate that raw food products play a minor role the colonization process. It was assumed that eating raw minced meat may promote the colonization of the human gut [14]. More interestingly, because of the high frequencies of isolation in turkeys and laying hens [3,13], the consumption and processing of poultry and eggs and its function as a transmission source from animals to humans should be focused on in following research perspectives and participants should be ask about their habits in terms of processing and eating chicken meat and eggs. At the beginning of this case-control study the high prevalence was not known. The transmission from poultry to humans is well-known for *Campylobacter*. The main significant risk factor for the transmission of this species to humans is particularly bought fresh chicken (OR 5.80; 95%-CI: 2.11–15.93), whereby it was decreased by eating fruit, raw vegetables, high-fiber cereals, vitamins and acidified milk products [50].

In summary, the case-control study conducted demonstrates a very high prevalence of *S*. *gallolyticus* subsp. *gallolyticus* in the gastrointestinal tract of healthy volunteers. In accordance with other researchers [8], it is essential to determine at least the subspecies or the biotype of the *S*. *bovis* strains to establish the identification of the frequency of *S*. *gallolyticus* in correlation with colorectal cancer, IE and other diseases, as well as its global impact to assess the risk to the human population. The data collected were evaluated with the help of multivariate statistical analyses to identify risk factors for the colonization of the human gut with the facultative pathogen. The simultaneous observation of exposure factors identified the closer contacts with animals and the usage of animals waste as significant risk factors for the detection of *S*. *gallolyticus* subsp. *gallolyticus* in human feces. Further investigations have to be performed to clarify the impact of chicken meat products and protective factors, such as vegetables. In addition, future studies should also include participants with e. g. gastrointestinal disorders associated with *S*. *gallolyticus* subsp. *gallolyticus* to detect the zoonotic pathogenicity of the Gram-positive bacterium to the health status of animals and humans and to determine the rate of *S*. *gallolyticus* subsp. *gallolyticus* fecal colonization. Another approach would be a prospective cohort study of people carrying *S*. *gallolyticus* subsp. *gallolyticus* or modifications of the gut microbiome along with environmental factors to detect the time-dependent influences on human health.

In addition to the detected potential risk factors, the vitality outside the gastrointestinal tract is also important for the direct or indirect transmission of the bacterium between animals and humans or between the environment and animals or humans, and should be pointed out in future studies.

## Supporting information

S1 Fig Relationship of 79 STs of *S*. *gallolyticus* subsp. *gallolyticus* isolates. Based on the allelic profile an Minimum spanning tree (MST) was constructed and clonal complexes (CC) were calculated by use of eBURST. CCs are presented as black lines. Each circle represents a ST and the size corresponds with number of bacterial isolates included. The STs of the case-control are presented as red dotted lines.
(TIF)

S1 Table Cross-tabulations of frequencies of potential risk factors observed and their distribution in cases and controls. (DOCX)
