# Supplementary figures and images for: Case-control study: Determination of potential risk factors for the colonization of healthy volunteers with Streptococcus gallolyticus subsp. gallolyticus
Source: PLoS One. 2017 May 1;12(5):e0176515. doi: 10.1371/journal.pone.0176515 (PMC5411088; doi:10.1371/journal.pone.0176515)

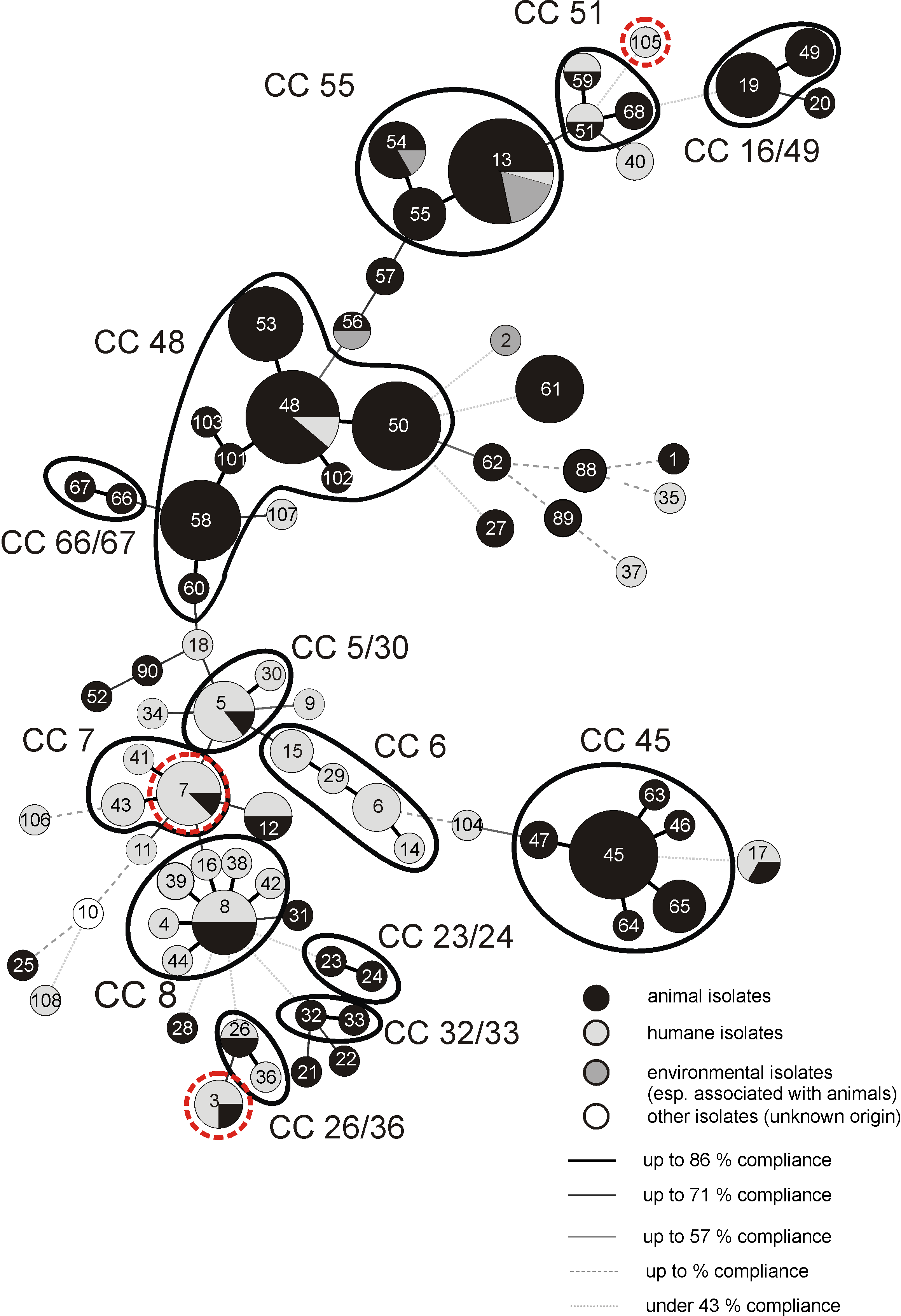

Supplement: S1 Fig — Based on the allelic profile an Minimum spanning tree (MST) was constructed and clonal complexes (CC) were calculated by use of eBURST. CCs are presented as black lines. Each circle represents a ST and the size corresponds with number of bacterial isolates included. The STs of the case-control are presented as red dotted lines. (TIF) [file pone.0176515.s001.TIF]
