# Supplementary material for: Case-control study: Determination of potential risk factors for the colonization of healthy volunteers with Streptococcus gallolyticus subsp. gallolyticus
Source: PLoS One. 2017 May 1;12(5):e0176515. doi: 10.1371/journal.pone.0176515 (PMC5411088; doi:10.1371/journal.pone.0176515)
Supplement: S1 Table — (DOCX) [file pone.0176515.s002.docx]

S1 Table: Cross-tabulations of frequencies of potential risk factors observed and their distribution in cases and controls.

| **exposure factor** | **Absolute frequency** | | |
| --- | --- | --- | --- |
|  |  | **Case** | **Control** |
| Age [years] ^#^ | >50 | 25 | 22 |
|  | <50 | 34 | 18 |
| Gender^#^ | male | 25 | 21 |
|  | female | 34 | 19 |
| Residence^#^ | urban | 40 | 22 |
|  | rural | 19 | 18 |
| Consumption of raw animal products^‡;#^ | yes | 44 | 26 |
|  | no | 15 | 14 |
| Only minced pork | yes | 13 | 7 |
|  | no | 46 | 33 |
| Only minced beef | yes | 0 | 1 |
|  | no | 59 | 40 |
| Pork, beef and mixed meat | yes | 28 | 14 |
|  | no | 31 | 26 |
| Only raw milk, raw milk products | yes | 3 | 4 |
|  | no | 56 | 36 |
| Processing of raw meat | yes | 50 | 30 |
|  | no | 9 | 10 |
| Professional/private contact with animals | yes | 38 | 23 |
|  | no | 21 | 17 |
| Pets | yes | 25 | 19 |
|  | no | 34 | 21 |
| Farm animal/livestock- | yes | 8 | 9 |
|  | no | 51 | 31 |
| Living or working on a farm | yes | 8 | 6 |
|  | no | 51 | 34 |
| Sporadic animal contact | yes | 18 | 22 |
|  | no | 41 | 18 |
| Factual animal contact | yes | 16 | 14 |
|  | no | 43 | 26 |
| Close animal contact^§, #^ | yes | 12 | 17 |
|  | no | 47 | 23 |
| Living next to a farm | yes | 18 | 17 |
|  | no | 41 | 23 |
| Living near the forest | yes | 22 | 23 |
|  | no | 37 | 17 |
| Vegetable growing | yes | 32 | 16 |
|  | no | 27 | 24 |
| Utilization of manure as fertilizer^#^ | yes | 20 | 5 |
|  | no | 39 | 35 |

^‡^ raw minced meat, raw milk and raw milk products; ^§^ Contact to excrement or saliva of animals, striking of animals; ^**^ variables used for the logistic regression model
